# Flexible and stretchable polymer optical fibers for chronic brain and vagus nerve optogenetic stimulations in free-behaving animals

**DOI:** 10.1186/s12915-021-01187-x

**Published:** 2021-11-24

**Authors:** Yi Cao, Suwan Pan, Mengying Yan, Chongyang Sun, Jianyu Huang, Cheng Zhong, Liping Wang, Lu Yi

**Affiliations:** 1 School of Life Sciences, Division of Life Sciences and Medicine, University of Science and Technology of China, Hefei, 230001 China; 2 Guangdong Provincial Key Laboratory of Brain Connectome and Behavior, CAS Key Laboratory of Brain Connectome and Manipulation, the Brain Cognition and Brain Disease Institute, Shenzhen Institute of Advanced Technology, Chinese Academy of Sciences, Shenzhen-Hong Kong Institute of Brain Science-Shenzhen Fundamental Research Institutions, Shenzhen, 518055 China

**Keywords:** Optogenetics, Flexible optical fibers, Brain stimulation, Vagus nerve stimulation, Anxiety-like behavior

## Abstract

**Background:**

Although electrical stimulation of the peripheral and central nervous systems has attracted much attention owing to its potential therapeutic effects on neuropsychiatric diseases, its non-cell-type-specific activation characteristics may hinder its wide clinical application. Unlike electrical methodologies, optogenetics has more recently been applied as a cell-specific approach for precise modulation of neural functions in vivo, for instance on the vagus nerve. The commonly used implantable optical waveguides are silica optical fibers, which for brain optogenetic stimulation (BOS) are usually fixed on the skull bone. However, due to the huge mismatch of mechanical properties between the stiff optical implants and deformable vagal tissues, vagus nerve optogenetic stimulation (VNOS) in free-behaving animals continues to be a great challenge.

**Results:**

To resolve this issue, we developed a simplified method for the fabrication of flexible and stretchable polymer optical fibers (POFs), which show significantly improved characteristics for *in vivo* optogenetic applications, specifically a low Young’s modulus, high stretchability, improved biocompatibility, and long-term stability. We implanted the POFs into the primary motor cortex of C57 mice after the expression of CaMKIIα-ChR2-mCherry detected frequency-dependent neuronal activity and the behavioral changes during light delivery. The viability of POFs as implantable waveguides for VNOS was verified by the increased firing rate of the fast-spiking GABAergic interneurons recorded in the left vagus nerve of VGAT-ChR2 transgenic mice. Furthermore, VNOS was carried out in free-moving rodents via chronically implanted POFs, and an inhibitory influence on the cardiac system and an anxiolytic effect on behaviors was shown.

**Conclusion:**

Our results demonstrate the feasibility and advantages of the use of POFs in chronic optogenetic modulations in both of the central and peripheral nervous systems, providing new information for the development of novel therapeutic strategies for the treatment of neuropsychiatric disorders.

## Background

The vagus nerve represents a main component of the peripheral nervous system (PNS) that bridges the central nervous system (CNS) and other organs. It has attracted increasing attention owing to its crucial role in regulating brain functions [1–4] and basic physiological responses, such as changes in the heart and respiratory rate [5, 6]. In addition to deep brain stimulation, vagus nerve electrical stimulation (VNES) is another frequently applied neural modulation strategy, which has shown remarkable therapeutic effects on several neuropsychiatric diseases, including epilepsy [7], anxiety [8], and depression [9]. However, the affected neuron populations and detailed manipulation mechanisms of VNES are poorly understood owing to the non-cell-type-specific activation characteristics of electrical stimulation. Therefore, novel intervention strategies are required for precise vagus nerve modulation in vivo.

Unlike electrical neural modulation methodology, optogenetics allows excitation or inhibition of specific types of neurons at a millisecond-level in free-behaving animals [10–13]. Besides, it prevents large electrical stimulus artifacts that may overlap the electrophysiological responses during recording. Due to those advantages, optogenetics has shown great potential for understanding the intrinsic mechanisms of various neuropsychiatric disorders [14–16] and developing cell-type-specific therapeutic intervention strategies [17–19]. To date, the most widely used implantable optical waveguides for optogenetics are silica optical fibers (SOFs), with an average Young’s modulus at least six orders of magnitudes higher than that of the neural tissues [20]. The huge elastic difference between the SOFs and organisms may lead to repeated injury and severe inflammatory responses to the host tissues, which subsequently induce neuronal death and tissue encapsulation in the implant surroundings [21]. To precisely manipulate the activities of specific neurons or neural circuits in particular brain regions, implantable SOFs are usually fixed on the skull bone to illuminate the light-sensitive protein expressed cells [22–24]. This strategy can provide a relatively stable interface between the optical implants and brain tissues that usually undergo negligible (or low) deformations in vivo, which has been applied in dissecting the complex neural processing mechanisms of neuropsychiatric diseases and behavioral-related phenomena at the functional level. However, the mechanical mismatch between the unstretchable stiff implants and highly deformable soft tissues is fatal for long-term effective light delivery in vivo. Consequently, chronic optogenetic stimulation, especially vagus nerve optogenetic stimulation (VNOS), in free-behaving animals is still a great challenging task.

To prevent this, flexible optoelectronics have been developed, providing tools for optogenetic stimulation of the CNS and sciatic nerve [25–28]. However, the complex design and fabrication process of these implantable devices may hinder their widespread application for various experimental and therapeutic purposes. Recent advances in polymer-based optical probes have provided an alternative strategy for flexible optogenetic modulations in freely behaving animals [29–32]; nevertheless, the stretchability of these optical implants (typically < 20%) may be inadequate to endure large tissue deformations, especially during chronic VNOS. To overcome this limitation, bio-stable hydrogel optical fibers have been developed, which exhibit a higher stretchability (100–500%) and lower Young’s modulus (~kPa) [21, 33–35]. However, their low refractive index (RI) and numerical aperture (NA) values may create an inconsistent performance in the complex biological environment, particularly in the cases of large bendings of the implanted optical fibers in vivo. Therefore, imparting flexible optical implants with highly stretchable optical waveguide characteristics for VNOS in free-moving animals requires further investigation.

In this study, we developed a simplified method for the fabrication of a flexible and stretchable core/clad structured PDMS/hydrogel polymer optical fiber (POF) and investigated the feasibility of its optogenetic applications in vivo. The optical and mechanical properties of the POF were evaluated, and the tissue response around the chronically implanted POF was studied. To determine whether the fabricated POF deliver enough light to activate the viral-expressing neurons in vivo, electrophysiological and behavioral responses before and during optogenetic stimulations were compared, respectively. Furthermore, the POF was implanted towards to the left vagal ganglion of VGAT-ChR2 transgenic mouse, and the effects of VNOS on animal physiology and behavior were systematically evaluated. All these results were analyzed and discussed against the requirements of flexible optogenetic applications in free-behaving animals.

## Results

### Fabrication and characterizations of POFs

**Fig. 1:**
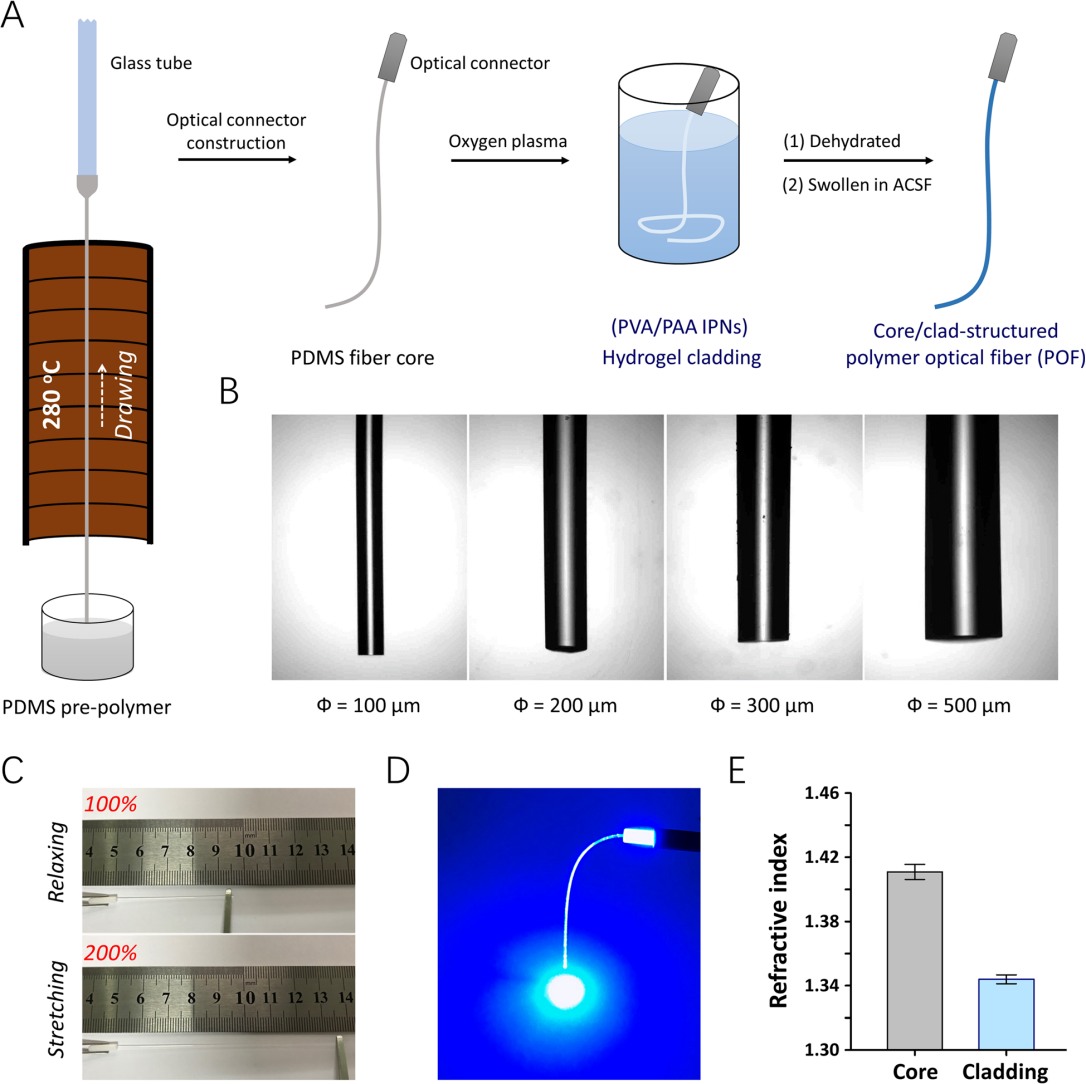
Fabrication and characterizations of polymer optical fibers (POFs). **A** A schematic illustration of the fiber core drawing process and the coating of the cladding layer. **B** Optical images of the POFs. **C** A representative example of a swollen POF before and during stretching. **D** A representative image of a POF exhibiting excellent light conductivity. **E** The refractive index of the core (PDMS) and cladding (PVA/PAA hydrogel) of the POF (*n* = 6 in each group, mean ± standard deviation)

To facilitate light transmission in the biological environment, the POF was composed of a core/clad structure. PDMS fibrous waveguides were constructed as the fiber cores via a thermal drawing process (Fig. 1A). As PDMS can be quickly cured (~ 2 s) at 280 °C, fiber cores with a wide range of feature dimensions (diameter = 100–500 μm) can be created at different pulling-up speeds of the pre-polymer (Fig. 1B). To balance the implantation trauma and optical performance of the POFs, fiber cores with a diameter of 200 μm were used in this study. Besides, poly(vinyl alcohol)/poly(acrylic acid) interpenetrating polymer network (PVA/PAA IPN) with a low RI of 1.3440 and a linear swelling ratio of 2.75 was synthesized as a cladding material (Additional file 1: Fig. S1 and S2A). Then, an oxygen plasma-treated PDMS fiber core was coated with the prepared PVA/PAA hydrogel solution, dehydrated, and re-swollen in an artificial cerebrospinal fluid (ACSF). Subsequently, a homogeneous hydrogel cladding layer was formed on the surface of the PDMS optical fiber core (Additional file 2: Fig. S2B).

To form a smooth optical connection for in vivo optogenetics, the fabricated POF was coupled with an optical ceramic ferrule and immersed in ACSF prior to use. The elastic POF showed a linear stress-strain relationship up to 150% strain and a Young’s modulus of 1.22 MPa (Additional file 3: Fig. S3A), which can steadily hold the stiff ceramic ferrule under 100% stretching deformation (Fig. 1C, Additional file 13: Movie 1). Four hundred seventy-two nanometers of blue laser light, commonly used for channelrhodopsin-2 (ChR2) excitation, was transmitted into the POF through a commercial SOF (diameter = 200 μm, NA = 0.37) terminated with a ceramic connector (Fig. 1D). To investigate the mechanical stability of those fabricated waveguides, we test the output power of POFs before and after repeated stretching to 200% of their original length for 10,000 times and found no significant influence on their optical transmission capability (Additional file 4: Fig. S4A). As the average RIs of the PDMS core (*n*_core_) and PVA/PAA cladding (*n*_clad_) were 1.4109 and 1.3440 respectively (*n* = 6, Fig. 1E), the theoretical numerical aperture (NA = [*n*_core_^2^ − *n*_clad_^2^]^0.5^) of the fabricated POF was 0.4293. In addition, the blue-light propagation loss of the POFs measured in air (*n*_*air*_ = 1.000) using a cutback technique [33, 35] was 1.018 dB·cm^−1^ (Additional file 5: Fig. S5). On comparison with the light powers obtained in air, it was observed that our POFs can retain approximately 90% of the optical transmission capacity (Additional file 6: Fig. S6) in water (*n*_*water*_ = 1.333, similar to the RI of brain tissues), which can meet the requirements of different application scenarios.

### Long-term biocompatibility and stability of POFs

**Fig. 2:**
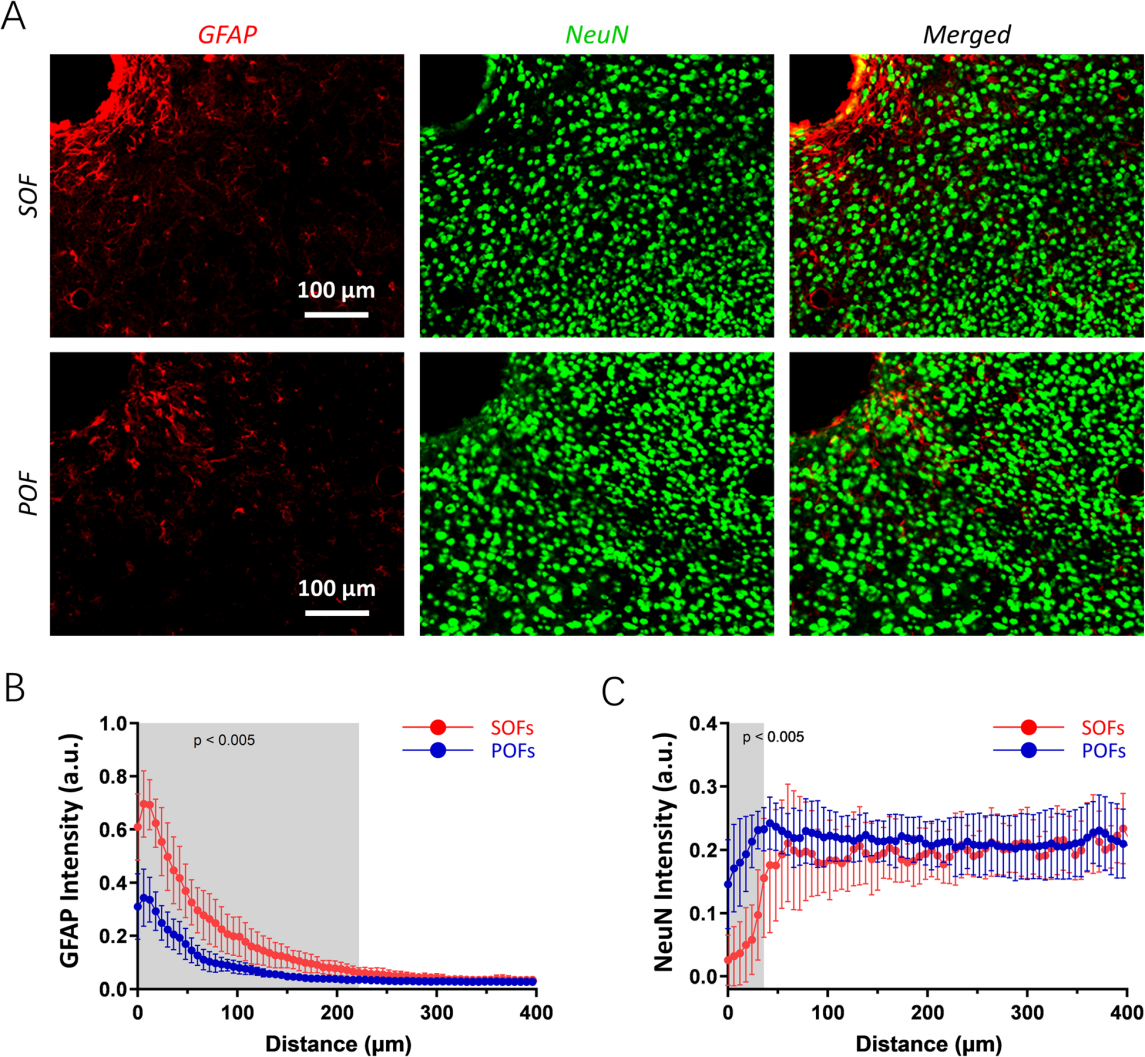
Histological characterization of the polymer optical fibers (POFs) in vivo. **A** Representative examples of GFAP (red) and NeuN (green) immunostaining of the conventional silica optical fibers (SOFs) and POFs at 4 weeks after implantation. **B**, **C** Quantitative comparisons of GFAP (**B**) and NeuN (**C**) immunoreactivity between the SOFs and POFs (*n* = 8 in each group, mean ± standard deviation). Comparisons were performed using intensity profiles as a function of distance from the implant interface (shaded area: *p* < 0.005, *t* test)

To examine the long-term biocompatibility of the POFs, samples were implanted into the brain of C57 mice for 4 weeks (Fig. 2A). Reactivated astrocytes occupied the zone around the SOF implants, whereas a considerably lighter glial fibrillary acidic protein (GFAP) positive zone was adjacent to the interface of the POFs. A quantitative analysis of the GFAP intensity as a function of the distance from the interface is presented in Fig. 2B, which shows that the GFAP intensity in the POF group was significantly lower than that of the SOF group (*n* = 8 mice, *p* < 0.005, *t* test), along 220 μm to the implant interface. Furthermore, the neuronal survival around the implants was assessed by analyzing the neuronal nucleus (NeuN) immunoreactivity. The neuronal density for the POF group was significantly higher than that of the SOF group in the test zone (*n* = 8 mice, *p* < 0.005, *t* test), within 40 μm of the implant-tissue interface (Fig. 2C). We also investigated the long-term physical stability of those fabricated waveguides. After the 4 weeks implantation, the Young’s modulus of the POF was 1.19 MPa, similar to the value before implantation (Additional file 3: Fig. S3B). Besides, no significant influence on the optical transmission capability of the POFs was observed after the long-term implantation (Additional file 4: Fig. S4B). These results suggest the long-term stability and validity of the fabricated POFs.

### Chronic optogenetic verifications

**Fig. 3.**
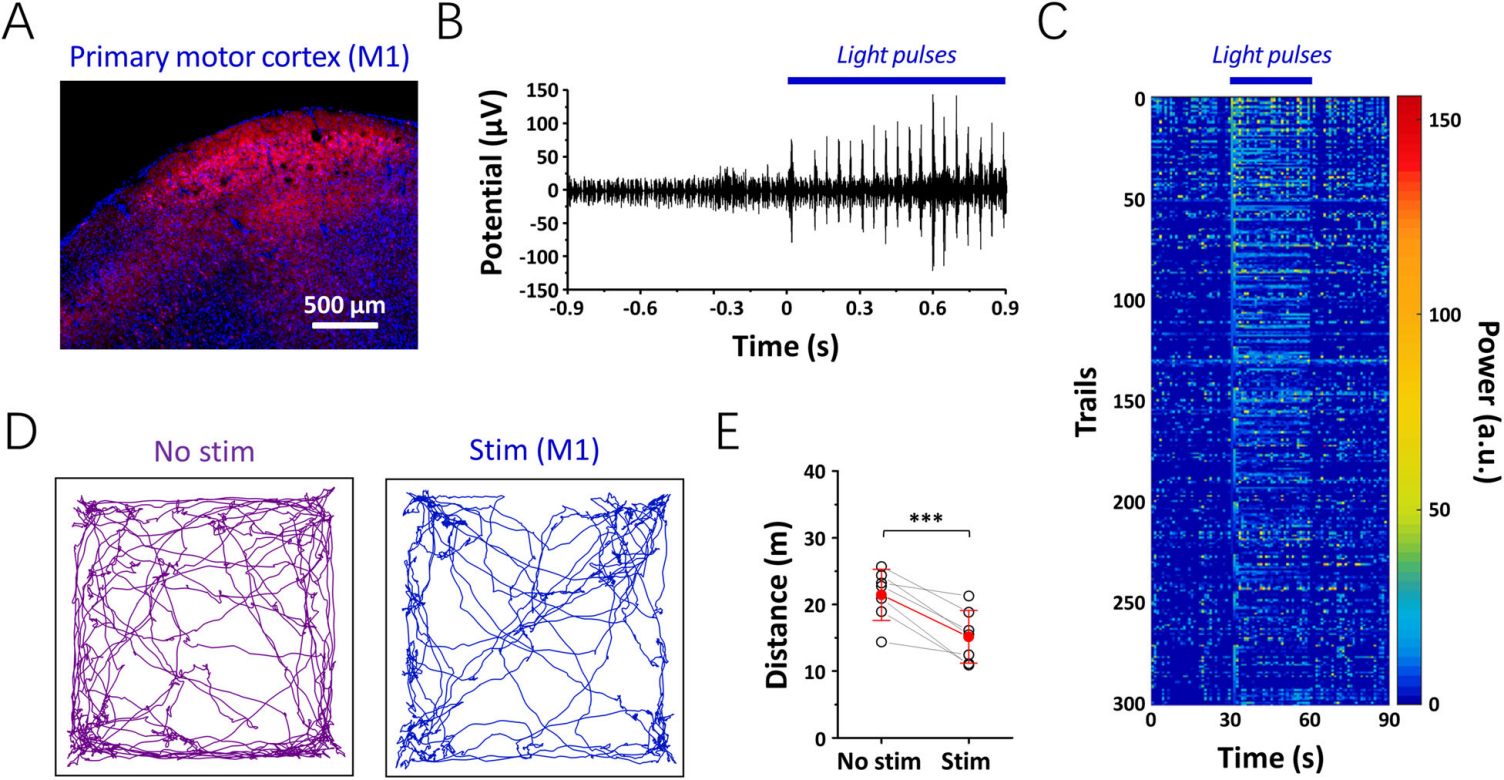
Optogenetic verification of the polymer optical fibers (POFs) in vivo. **A** The expression of CaMKIIα-ChR2-mCherry (red) in the primary motor cortex (M1) 4 weeks after viral injection. **B** Representative example of electrophysiological recordings before and during optogenetic stimulation. **C** Neurogram in response to optogenetic stimulations (472 nm, 20 Hz, 5 ms duration). **D** Representative moving traces in an open field before and during optogenetic modulation. **E** The total distance traveled in the open field is shown (*n* = 7 mice, mean ± standard deviation; ***p < 0.005, paired *t* test)

To determine whether the POFs could deliver enough light for optogenetic modulations in vivo, adeno-associated virus (AAV)-CaMKIIα-ChR2-mCherry was injected into the hippocampus of a C57 mouse (Additional file 7: Fig. S7). After the viral expression, a custom-made optrode array containing a POF and two tetrodes was used for in vivo optogenetic stimulation and electrophysiological recording. Continuous, frequency-dependent action potentials were detected when light pulses (472 nm, 20 Hz, 5 ms duration) were delivered to the hippocampus, suggesting that the fabricated POFs can serve as waveguides for in vivo optogenetic stimulation. To further investigate the long-term validity of these optical waveguides, POFs were implanted into the primary motor cortex (M1) 1 week after AAV-CaMKIIα-ChR2-mCherry injection (Fig. 3A). Four weeks after viral injection, blue light pulses (20 Hz, 5 ms duration) were conducted through the implanted POFs to activate the ChR2-expressing neurons in the M1, and continuous action potentials of the optically activated neurons were recorded (Fig. 3B, C). Interestingly, we also observed that the optogenetic stimulation caused motor coordination impairment in the mice (Fig. 3D), including increased turning, rotating, and falling behaviors. Consequently, the total moving distance during M1 activation (15.15 ± 3.96 m) was significantly decreased (*n* = 7, *p* < 0.005, *t* test) compared to the behavioral results obtained on the mice before optogenetic stimulation (21.46 ± 3.83 m) (Fig. 3E).

### Effect of VNOS on physiological responses

**Fig. 4.**
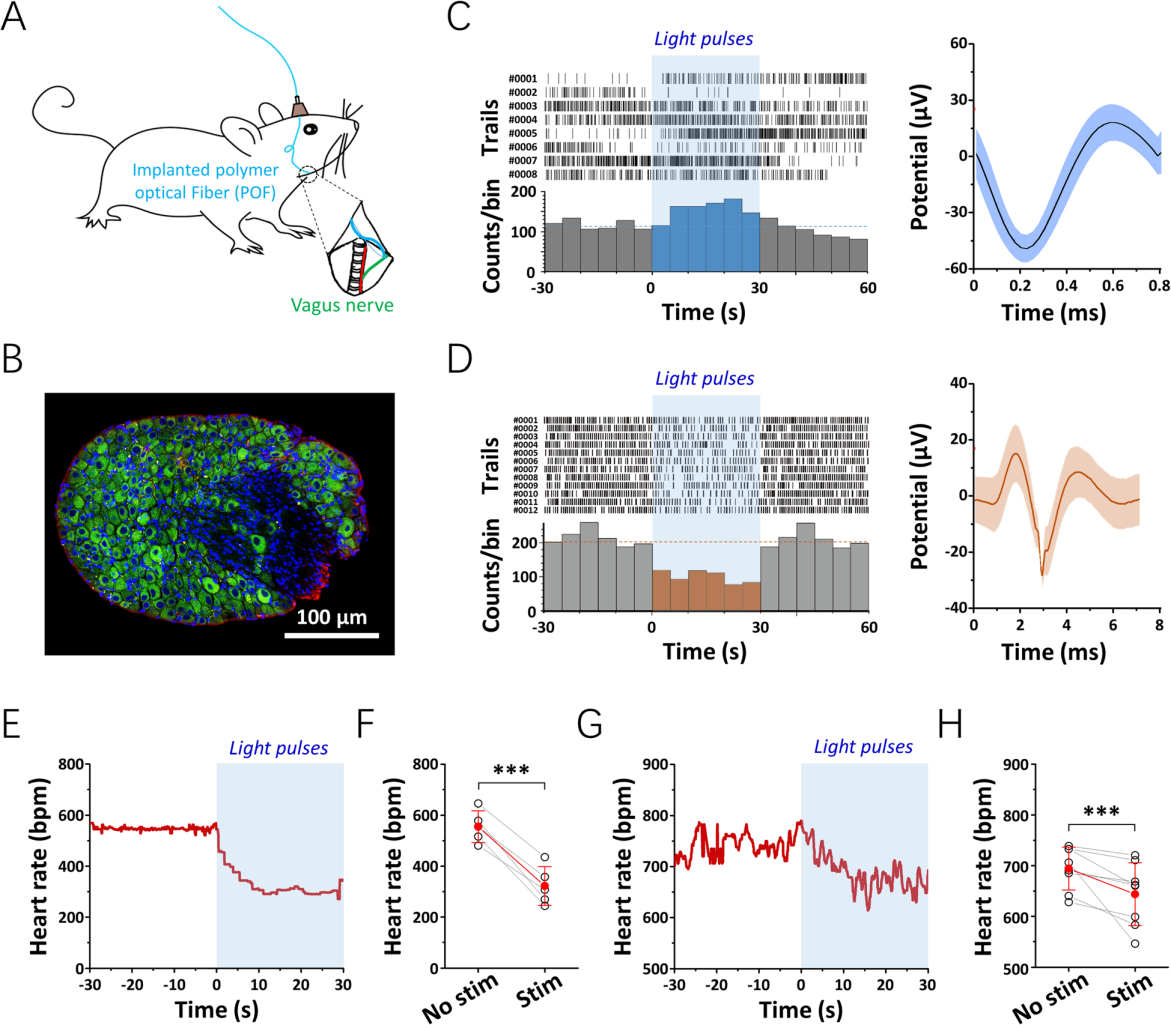
Optogenetic modulation of the vagus nerve in vivo. **A** A schematic illustrating the implantation of a polymer optical fiber (POF) for vagus nerve optogenetic stimulation (VNOS). **B** A representative image of the ganglion of the left vagus nerve is shown: blue, DAPI; green, VGAT; red, ChAT. **C**, **D** Representative raster plots of spike timestamps (top) and histogram plots of the firing rate (bottom) before, during, and after VNOS are shown (**C**, putative GABAergic interneurons: spike duration < 1 ms; **D**, putative cholinergic neurons: spike duration > 6 ms). **E**–**H** Representative examples (**E**, **G**) and quantitative analyses (**F**, **H**) of the heart rate variability in anesthetized (**E**, **F**; *n* = 5 in **F**, mean ± standard deviation) and freely behaving (**G**, **H**; *n* = 8 in **H**, mean ± standard deviation) mice (***p < 0.005, paired *t* test). The shaded area indicates the VNOS period

To study the feasibility of using our fabricated waveguide for chronic VNOS in free-moving rodents, we implanted the POF under the mouse skin, and fixed the ceramic ferrule connector to the skull with dental cement for in vivo optogenetic stimulation (Fig. 4A). The tip of the POF was placed towards to the left vagal ganglion of VGAT-ChR2 transgenic mouse and fixed in place with 3M tissue glue to construct a smooth and stable optical connection (Additional file 8: Fig. S8). A micro-wire stereotrode was implanted in the vagal ganglion to record the electrophysiological responses during light delivery (472 nm, 130 Hz, 5 ms duration). We found that VNOS selectively activated the fast-spiking γ-aminobutyric acid (GABA)-ergic interneurons, while suppressed the broad-spiking cholinergic neurons (Fig. 4B–D).

Furthermore, the heart rates (HRs) of the mice were monitored to investigate the influence of VNOS on innate physiological responses. It is worth mentioning that, as the frequency for the VNOS exhibits a dose-dependent inhibitory effect on the cardiac activity of the VGAT-ChR2 transgenic mouse, 130 Hz was chosen as an optimized stimulation frequency for the modulation of the vagus nerve in this study (Additional file 9: Fig. S9). Under anesthesia, the average HR of the mice was 555.5 ± 62.7 beats per minute (bpm), which dramatically decreased (*n* = 5, *p* < 0.005, *t* test) during optical stimulation and reached a plateau of 322.7 ± 76.4 bpm (Fig. 4E, F). To investigate the validity and reliability of the POFs for optogenetic stimulation under spontaneous tissue deformations, we monitored the HR in freely behaving animals before and during VNOS. The HRs recorded in awaken mice were higher than those obtained in anesthetized mice, with an average value of 694.4 ± 42.3 bpm. During light delivery, the average HR of the experimental subjects was significantly decreased (*n* = 8, *p* < 0.005, *t* test) to 643.8 ± 62.0 bpm (Fig. 4G, H).

### Effect of VNOS on behaviors

**Fig. 5.**
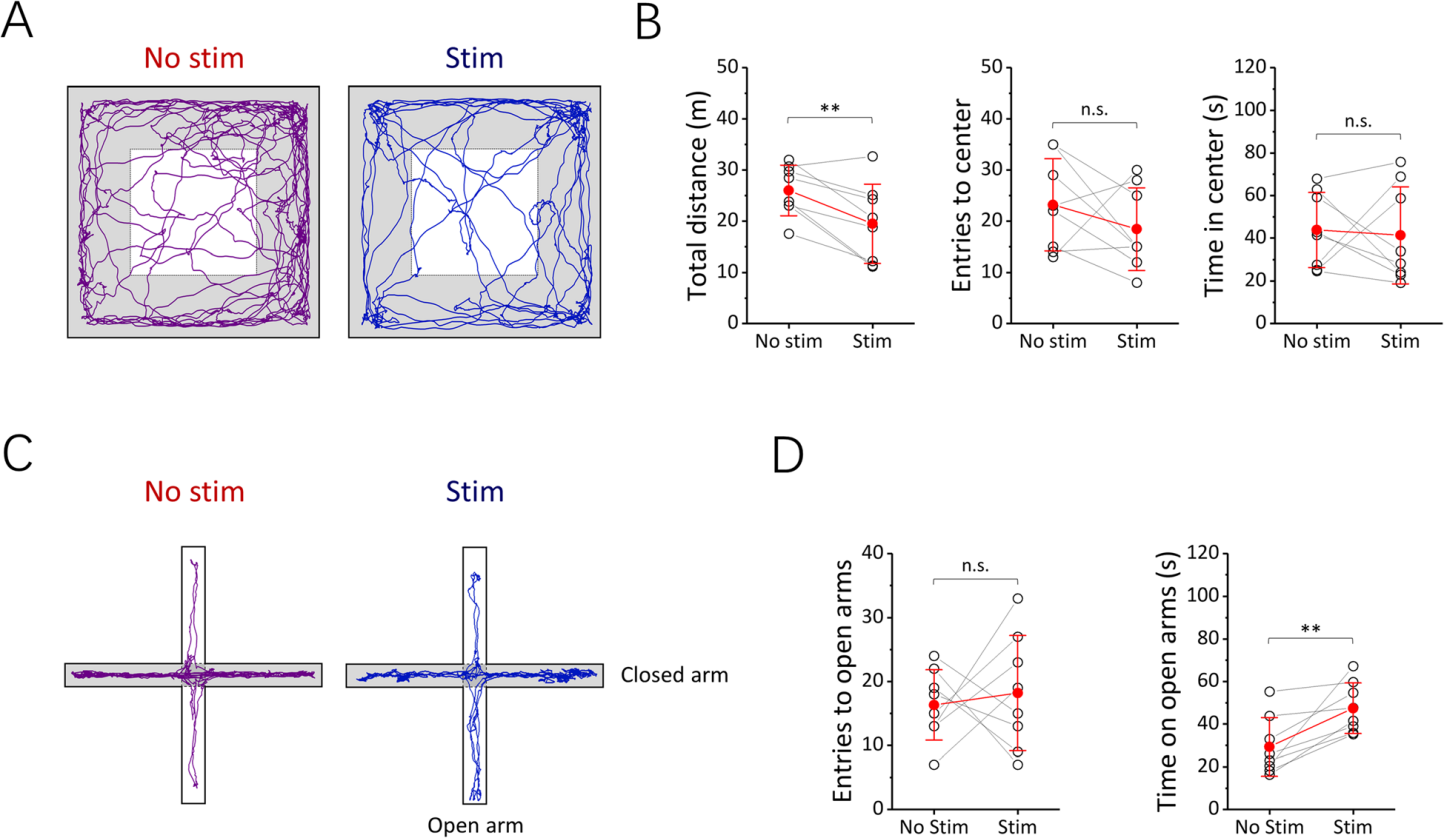
Effect on the behaviors of VGAT-ChR2 transgenic mice under vagus nerve optogenetic stimulation (VNOS). **A** The representative moving traces of the mice in an open field arena (OFA) before and during VNOS. **B** The total distance traveled in the OFA, entries to the center zone and time spent in the center zone (*n* = 8) of the OFA before and during VNOS. **C** The representative moving traces of the mice in an elevated plus maze (EPM) before and during VNOS. **D** Entries to and time spent on the open arms of the EPM (*n* = 8) before and during VNOS. The data are presented as mean ± standard deviation of the mean. The stars represent significant differences (**p < 0.01, paired *t* test)

To study the influence of VNOS on animal behaviors, we performed an open field test after POF implantation (Fig. 5A). We observed that the total distance traveled in the open field arena (OFA) reduced (*n* = 8, *p* < 0.01, *t* test) from 26.03 ± 4.96 m to 19.53 ± 7.73 m during optogenetic stimulation, while the total entries to and time spent in the center zone of the OFA were not significantly changed (*n* = 8, *p* > 0.05, *t* test) (Fig. 5B). To further investigate the effect of VNOS on emotion-related behaviors in mice, we then performed elevated plus-maze (EPM) test (Fig. 5C). Although optogenetic stimulation showed no significant influence (*n* = 8, *p* > 0.05, *t* test) on the open arm entries of the experimental subjects, the time spent on the open arms increased (*n* = 8, *p* < 0.01, *t* test) from 29.48 ± 13.71 s to 47.58 ± 11.87 s (Fig. 5D). In addition, we also monitored the HRs of experimental subjects during EPM test. We observed that the HRs increased (*n* = 4, *p* < 0.05, *t* test) from 677.59 ± 34.29 bpm to 711.29 ± 47.45 bpm when the mice moved from the closed arm to the open arm, which instantly dropped back to normal level (668.83 ± 62.87, *n* = 4) under vagal modulations (Additional file 10: Fig. S10).

**Fig. 6:**
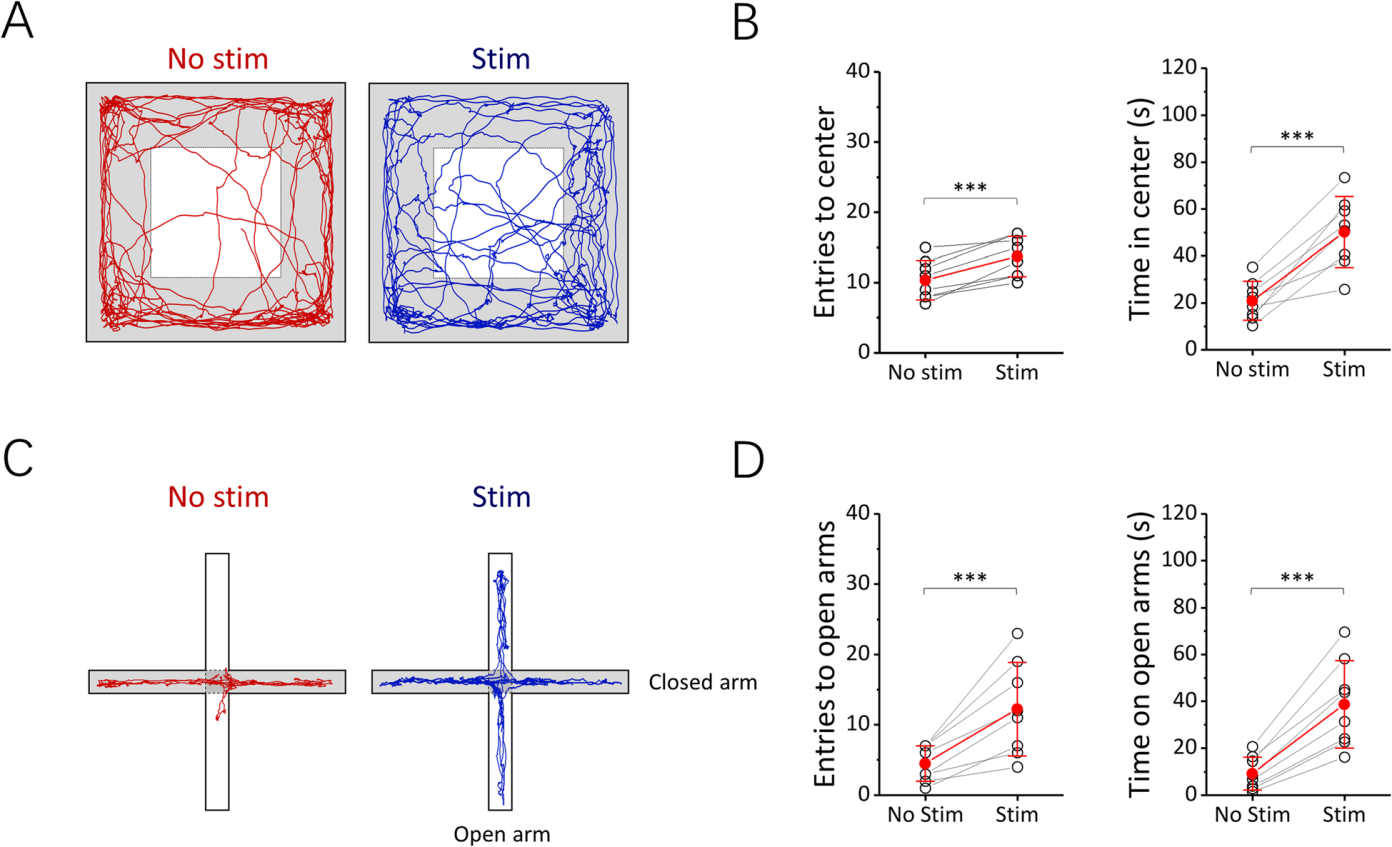
Effect on the behaviors of unpredictable chronic mild stress (UCMS)-treated VGAT-ChR2 transgenic mice under vagus nerve optogenetic stimulation (VNOS). **A** The representative moving traces of the mice in an open field arena (OFA) before and during VNOS. **B** Entries to and time spent in the center zone (*n* = 8) of the OFA before and during VNOS. **C** The representative moving traces of the mice in an elevated plus maze (EPM) before and during VNOS. **D** Entries to and time spent on the open arms of the EPM (*n* = 8) before and during VNOS. The data are presented as mean ± standard deviation of the mean. The stars represent significant differences (***p < 0.005, paired *t* test)

Next, we adopted an unpredictable chronic mild stress (UCMS) model to further investigate the effect of VNOS on animals’ behaviors. After the UCMS treatment, the total entries to the center zone of the OFA decreased (*n* = 4, *p* < 0.05, *t* test) from 27.00 ± 7.16 times to 10.00 ± 2.94 times, and the time spent in the center zone decreased (*n* = 4, *p* < 0.05, *t* test) from 69.45 ± 27.20 s to 21.05 ± 5.65 s (Additional file 11: Fig. S11A, B). Besides, the open arm entries of the animals decreased (*n* = 4, *p* < 0.05, *t* test) from 11.00 ± 6.98 times to 4.00 ± 2.94 times, and the time spent on the open arms also decreased (*n* = 4, *p* < 0.01, *t* test) from 41.38 ± 15.19 s to 7.15 ± 5.70 s (Additional file 11: Fig. S11C, D). Then, we investigated the effect of VNOS on the anxiety-like behaviors of the experimental subjects. Interestingly, during VNOS, the total entries to the center zone of the UCMS-treated animals increased (*n* = 8, *p* < 0.005, *t* test) from 10.38 ± 2.83 times to 13.75 ± 2.87 times, and the time spent in the center zone increased (*n* = 8, *p* < 0.005, *t* test) from 21.03 ± 8.31 s to 50.30 ± 15.11 s (Fig. 6A, B). Besides, the open arm entries of the animals increased (*n* = 8, *p* < 0.005, *t* test) from 4.50 ± 2.51 times to 12.25 ± 6.67 times, and the time spent on the open arms increased (*n* = 8, *p* < 0.005, *t* test) from 9.38 ± 6.99 s to 38.83 ± 18.68 s (Additional file 11: Fig. S11C, D).

Furthermore, to study the long-term influence of VNOS, behaviors of the UCMS-treated animals were monitored each week. After repeated VNOS treatments, we found that the animals exhibited an increased tendency to explore the center zone of the OFA and the open arms of the EPM, respectively (Additional file 11: Fig. S11). In addition, after long-term VNOS, tissue response around the POFs was studied using hematoxylin-eosin (HE) staining, and no significant cell loss (*n* = 30 slices from 5 mice, *p* > 0.05, *t* test) was observed in the POF-contacted tissues (Additional file 12: Fig. S12). It proves the mechanical compatibility between the stretchable POFs and the soft tissues and implies that the POFs can meet the requirement of flexible optogenetic applications in vivo.

## Discussion

In this study, we systematically investigated the feasibility and validity of a flexible POF for chronic optogenetic stimulations in freely behaving animals. To guarantee the efficiency and stability of light transmission, a core/clad structured polymer optical fiber was fabricated, with a theoretical NA value of 0.4293, comparable to that of the commercial SOF. Benefited from its excellent optical characteristics, we observed a relatively low blue-light propagation loss of the fabricated POFs in different mediums. Besides, as the fiber cores within a wide diameter range can be obtained at different pulling-up speeds of the pre-polymer during the thermal drawing process, the diameter size of our optimized POFs (total diameter ≈ 250 μm) was much smaller than that of the reported core/clad-structured hydrogel optical fibers (diameter = 920–2200 μm) [35]; thus, it is more suitable for optical delivery to the brain and the vagus nerve in vivo.

We first investigated the feasibility of a POF use for chronic BOS in free-moving animals. In comparison with the commercialized SOFs, we observed a decreased glial encapsulation and an improved neuronal viability around the POFs after long-term implantation. It implies an excellent biocompatibility of the POFs, which may be attributed to the improved mechanical compatibility between the flexible POFs and the soft neural tissues, as well as the neural-friendly characteristic of the POF interface. Attributed to these merits, we successfully constructed a smooth and stable optical connection for BOS in vivo and recorded frequency-dependent action potentials during light delivery. Besides, we found that optogenetic stimulation through the implanted POFs in the M1 can considerably modulate the behavior of mice, suggesting that these optical waveguides can transmit sufficient light power to activate the targeting neurons after long-term implantation. Altogether, owning to their excellent biocompatibility and optical transmission0 capacity, these POFs are suitable for chronic optogenetic modulations in vivo and provide novel insights in future therapies of neuropsychiatric disorders.

Furthermore, it is worth mentioning that the huge mechanical mismatch between the optical implants and organisms may cause severe neural injury and inflammatory responses in free-behaving animals. The conventional SOFs are especially not suitable for VNOS, as the tissue deformations during spontaneous body movement can lead to repeated displacement of the stiff implant and severe injuries of the surrounding tissues. In this work, the Young’s modulus of our POF is similar to that of biological tissues, which is four orders of magnitude lower than that of the conventional SOFs (~10 GPa). The strain at break of the POFs is higher than 150%, implying a superior stretchability compared to the reported polymer-based optical implants for in vivo optogenetics [29, 36]. In addition, we did not observe noticeable cracks or significant influence on the output power of the POFs after repeated 100% stretching deformation, which implies an excellent mechanical stability of the fabricated optical waveguides [37–39]. Those advantages greatly expand the potential applications of optogenetics and enable optical delivery in soft tissues even with large deformations.

To investigate the validity of the POFs in practical use, we implanted the POF towards to the left vagal ganglion of VGAT-ChR2 transgenic mouse for VNOS. We observed that light delivery to the vagus nerve increased the firing rate of the fast-spiking GABAergic interneurons, which implies that the POFs can overcome the contradiction of mechanical mismatch between the optical implants and soft organisms and serve as waveguides for VNOS in vivo. Interestingly, we observed that the HRs of the experimental subjects significantly decreased during VNOS, suggesting an inhibitory effect on innate physiological responses. Besides, we found that the HRs of mice were significantly increased when the experimental subjects moved from the closed arm to the open arm, which instantly dropped back to normal level during VNOS. This implies that the VNOS may diminish the elevated physiological arousal level under negative stimuli. It should be noted that, the fluctuation of the HRs (< 10%) obtained from the freely behaving mice during VNOS is within the range of physiological responses under natural conditions, it will not lead to a fatal response of the cardiac system.

We next performed open field and EPM tests to study the influence of VNOS on the behaviors of animals. An anxious state was induced after the UCMS treatment, evidenced by the significantly decreased tendency of animals to explore the center zone of the OFA and the open arms of the EPM, respectively. However, VNOS significantly increased the entries to and time spent in the center zone of the OFA as well as the entries to and time spent on the open arms of the EPM, implying an inhibitory effect on the anxiety-like behaviors of the experimental subjects. Furthermore, we also observed a prolonged anxiolytic-like behavior after long-term vagal modulation, which is in accordance with recent studies that have used VNES [40]. As the vagus nerve sends multi-synaptic projections to brain regions associated with anxiety, including the locus coeruleus, hippocampus, and amygdala [41, 42], our findings imply that the GABAergic interneurons in the vagus nerve may have contributed to the neural circuits in anxiety regulation. These results reveal the long-term validity and anxiolytic effect of the VNOS, which confirms the functional effectiveness and stability of the fabricated POFs in freely behaving animals.

## Conclusion

In conclusion, we demonstrated the feasibility and advantages of a core/clad structured PDMS/hydrogel POF use for chronic brain and vagus nerve optogenetic stimulations in freely behaving animals. The PDMS core of the POF was fabricated via a thermal drawing process, and a hydrogel film was applied as a cladding layer. The fabricated POFs exhibited a low-modulus and high stretchability, facilitating their biocompatibility and mechanical stability with neural tissue especially under large deformations. The POFs also exhibited a high NA value and low blue-light propagation loss, and their validity as waveguides for in vivo applications was verified by the frequency-dependent electrophysiological responses during the optogenetic stimulation of hippocampal and M1 neurons. In addition, activation of the AAV-expressing neurons in M1 showed a significant influence on the animal behavior, evidenced by the impairment of motor coordination and significantly decreased moving distance. Because of the merits of the POFs, we performed VNOS and observed the light-evoked responses of different types of neurons in the vagus nerve. Light delivery through the chronic implanted POFs can activate the GABAergic interneurons in the vagus nerve, which directly reduced the physiological arousal level (decreased heart rate) of the experimental subjects and exhibited a prolonged anxiolytic-like behavior. The characteristics evaluated and discussed were based on the requirements of flexible optical waveguides for optogenetic applications in vivo. Besides, this approach has also shown great potential for cell-type-specific stimulation of neural tissue with large deformations, including the vagus nerve, spinal cord, sciatic nerve, and other organs, which may expand the applications of optogenetics and provide novel insights into future therapeutic treatments of neuropsychiatric disorders. The functional characteristic of those neural circuits is complex and the underlying mechanism remains largely unclear; thus, further systematic investigations are still required.

## Methods

### Fabrication and characterizations of POFs

The pre-polymer and curing agent of silicone elastomer (Dow Corning, Sylgard 184) were mixed at a ratio of 10:1 and then thoroughly degassed under a vacuum at room temperature prior to use. A glass tube was immersed into the mixed pre-polymer and pulled up through a tubular heater (length = 40 cm, temperature = 280 °C) to fabricate a polydimethylsiloxane (PDMS) fiber core (Fig. 1A). The diameter of the PDMS fiber cores can be controlled by the pulling-up speed of the pre-polymer. A poly(vinyl alcohol)/poly(acrylic acid) interpenetrating polymer network (PVA/PAA IPN) film was introduced as the cladding of a POF, which was synthesized following our previous works [10, 43]. Briefly, an appropriate weight of the acrylic acid monomer (Sigma-Aldrich) was added to the PVA solution (5 wt%, Sigma-Aldrich) under magnetic stirring at the desired ratios (molar ratio: 0.5, 1.0, 1.5, 2.0 of AA monomer per vinyl alcohol repeating unit). Ammonium persulfate (Degussa-AJ) was added at 1000 ppm as an initiator, and then the mixed solution was purged with argon for 15 min and allowed to react at 80 °C for 72 h in the absence of oxygen. The PVA/PAA samples were fully swollen in ACSF at room temperature before testing. The refractive index of the PDMS and PVA/PAA films were measured using an Abbe refractometer. To form a smooth optical connection for optogenetic stimulations, the PDMS fiber core was threaded through and coupled with an optical ceramic ferrule. After that, a thin layer of PVA/PAA IPNs was coated onto the surface of the oxygen-plasma treated PDMS core as a cladding. The fabricated POF was dehydrated at 80 °C for 2 h and immersed in ACSF prior to use.

### Chronic tissue response assessment

All experiments were performed in accordance with the protocols approved by the Ethics Committee for Animal Research, Shenzhen Institute of Advanced Technology, Chinese Academy of Sciences (SIAT-IACUC-200218-NS-LY-A1039). All animals were anesthetized using isoflurane (5% induction followed by 1.5–2.0%) and warmed with a heating pad during surgery. Conventional SOFs and fabricated POFs were implanted into the brain (AP − 2.06 mm, ML − 1.35 mm) of 12-week-old C57BL/6J mice, respectively. At 4 weeks after implantation, the mice were sacrificed and 35 μm thick horizontal sections were prepared. Fluorescence images were obtained using an Olympus VS120 microscope. Quantitative analyses were performed using a custom software developed in MATLAB (MathWorks, USA). The staining intensity of glial fibrillary acidic protein (GFAP; 1:1000; Abcam, Ab7260; RRID: AB_305808) and neuronal nucleus (NeuN; 1:500; Millipore, MAB377; RRID: AB_2298772) were calculated as a function of distance to the implant surface. The results shown represent the average intensity profiles of the analyzed area within 400 μm from the implant/tissue interface.

### Optogenetic verifications

Twelve-week-old C57BL/6J male mice were used for hippocampal and primary motor cortex (M1) modulations. Four weeks prior to testing, AAV9-CaMKIIα-ChR2-mCherry was injected into the hippocampus (AP − 2.06 mm, ML − 1.35 mm, DV − 2.0 mm) and M1 (AP +1.98 mm, ML − 1.80 mm, DV − 1.5 mm), respectively. To investigate the feasibility of the fabricated POF for in vivo optogenetic stimulation, the fiber was coupled with a custom-made tetrode array and then lowered into the hippocampus and M1, respectively. The tetrodes were fabricated from 12.5 μm diameter formvar-coated nickel chromium wires (A&M systems), which were electrodeposited with platinum nanoparticles prior to use (1 kHz impedance in ACSF < 500 kΩ). Electrophysiological recordings were obtained using a multi-channel neural acquisition processor (Plexon). The data were sampled at 40 kHz and bandpass filtered at 300–5000 Hz. To investigate the effect of optical modulation on the animal behavior, a POF was implanted into M1 and fixed to the skull with dental cement. The test mouse was allowed to freely move in an open field chamber (60 × 60 × 50 cm) for 10 min, and then 472-nm light pulses (10 mW, 20 Hz, 5 ms duration) were delivered into M1 through the implanted POF for an additional 10 min. The behaviors of the experimental subjects were recorded with a digital video camera and analyzed by the ANY-maze video tracking system (Stoelting). To verify the expression of AAV, 35 μm thick coronal cortical slices were obtained using a cryostat microtome (Leica CM1950), and ChR2-mCherry expressing hippocampal and M1 neurons were observed under an Olympus VS120 microscope.

### Optogenetic modulation of the vagus nerve

Twelve-week-old VGAT-ChR2 (H134R)-EYFP transgenic mice were used in the VNOS studies. Mice were anesthetized using isoflurane (5% induction followed by 2%) and placed on a surgical platform. The left vagus nerve was targeted to minimize the descending modulation effects on the sinoatrial node. Hair over the mouse neck area was removed, and then a vertical incision was made on the dorsal left side. Thereafter, the flexible POF tip was placed towards to the ganglion of the vagus nerve and fixed in place with a small amount of tissue glue (3M). To ensure a stable optical connection with the vagus nerve after long-term implantation, the front part of the POF was fixed to the surrounding muscle with nylon suture and tissue glue. The rest of the parts of the POF was implanted under the skin, and the ceramic ferrule (the end of the POF) was fixed to the skull with dental cement. The animals recovered for one week before further tests. To verify the feasibility of the POFs for use in VNOS in vivo, a stereotrode was inserted into the ganglion of the vagus nerve to obtain electrophysiological recordings during optogenetic stimulation (472 nm, 10 mW, 130 Hz, 5 ms duration). The stereotrode was fabricated from 12.5 μm diameter formvar-coated nickel chromium wires (A&M systems) and electrodeposited with platinum nanoparticles prior to use (1 kHz impedance in ACSF < 500 kΩ). To investigate the influence of VNOS on the animal physiological functions, the HR was monitored with a MouseOx system before and during optical stimulation (472 nm, 10 mW, 130 Hz, 5 ms duration).

We used UCMS-treated mice to investigate the effect of VNOS in the regulation of animal behaviors under anxious states. The UCMS protocol was performed following previously works [44, 45]. Mice were exposed to environmental stressors for two weeks. One of the following stressors were randomly presented during each day: (a) tight squeeze for 4 h, where each mouse was placed in a small sturdy box (3 cm × 5cm × 7 cm); (b) wet environment for 12 h, where water was added to a housing cage to wet the bedding; and (c) high-elevation stress for 1 h, where mice were kept on a 1-m-high platform. To study the effect of VNOS on the animal behaviors, an open field test (10 min light off, 10 min light on) and elevated plus-maze test (5 min light off, 5 min light on) were performed. The behaviors were recorded with a digital video camera and analyzed using the ANY-maze video tracking system (Stoelting). To verify the expression of VGAT-ChR2, 30-μm-thick slices of the vagal ganglion were obtained using a cryostat microtome (Leica CM1950) and observed under a laser scanning confocal microscope (LSM880, Zeiss).

## Data Availability

All data generated or analyzed during this study are included in this published article and supplementary information files. The raw data in this study are available from figshare (10.6084/m9.figshare.16908466.v1).

## Abbreviations

AAV: Adeno-associated virus
ACSF: Artificial cerebrospinal fluid
bpm: Beats per minute
BOS: Brain optogenetic stimulation
CNS: Central nervous system
ChR2: Channelrhodopsin-2
EPM: Elevated plus-maze
GFAP: Glial fibrillary acidic protein
HRs: Heart rates
HE: Hematoxylin-eosin
NeuN: Neuronal nucleus
NA: Numerical aperture
OFA: Open field arena
PNS: Peripheral nervous system
PVA/PAA IPN: Poly(vinyl alcohol)/poly(acrylic acid) interpenetrating polymer network
PDMS: Polydimethylsiloxane
POF: Polymer optical fiber
M1: Primary motor cortex
RI: Refractive index
SOFs: Silica optical fibers
POFs: Stretchable polymer optical fibers
UCMS: Unpredictable chronic mild stress
VNOS: Vagus nerve optogenetic stimulation
GABA: γ-Aminobutyric acid
